# Supplementary material for: Joint single-cell DNA accessibility and protein epitope profiling reveals environmental regulation of epigenomic heterogeneity
Source: Nat Commun. 2018 Nov 2;9:4590. doi: 10.1038/s41467-018-07115-y (PMC6214962; doi:10.1038/s41467-018-07115-y)
Supplement: Supplementary file 1 — Supplementary Information [file 41467_2018_7115_MOESM1_ESM.pdf]

Joint single-cell DNA accessibility and protein epitope profiling reveals environmental regulation of epigenomic heterogeneity

Chen et al.

Supplementary information

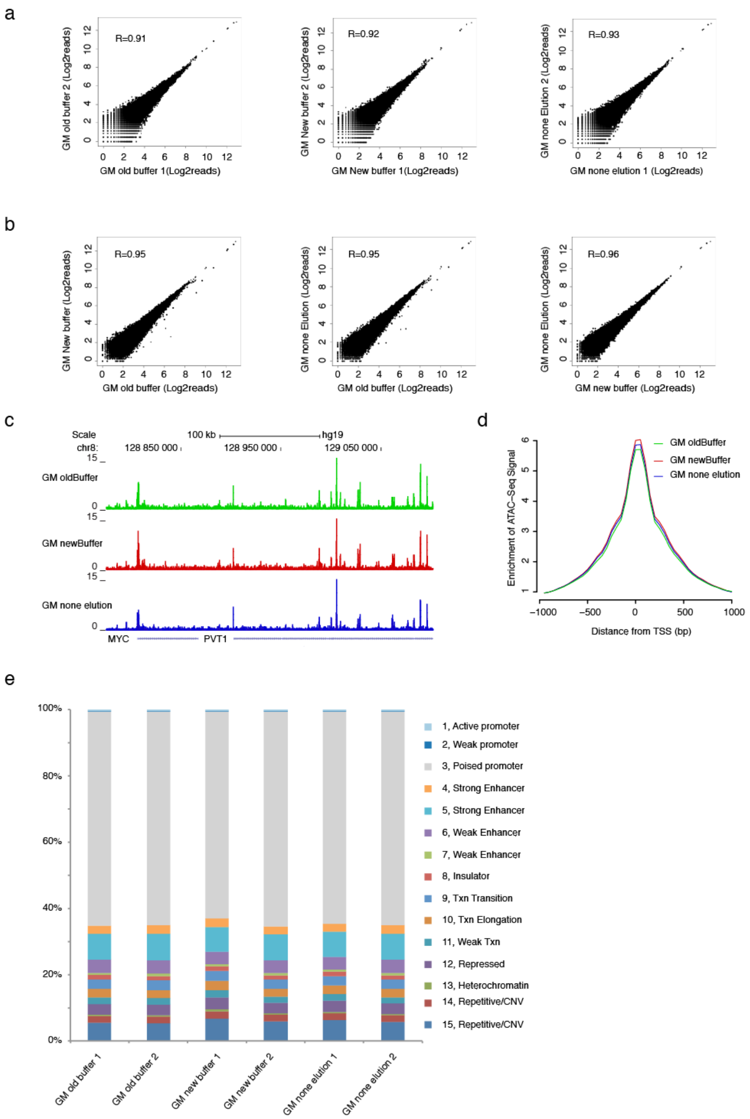

Supplementary Figure 1: Development of bulk Pi-ATAC. **a,** Left: Correlation of reads in peaks (log2) of ATAC-seq library replicates of fixed GM12878 cells, which were reverse-crosslinked using the old buffer [1] and then purified. Middle: Correlation of reads in peaks (log2) of ATAC-seq library replicates of fixed GM12878, which were reverse-crosslinked using a new buffer and then purified. Right: Correlation of reads in peaks (log2) of ATAC-seq library replicates of fixed GM12878, which were reverse-crosslinked using the new buffer and then not purified but directly barcoded by PCR, representing the bulk Pi-ATAC workflow; **b,** Comparison across fixed ATAC-seq methods. Left: Scatter plot of fragment quantification in peaks (log2) of fixed GM12878 using the old reverse crosslink buffer vs. new buffer. Middle: Scatter plot of fragment quantification in peaks (log2) of fixed GM12878 using the old reverse crosslink buffer vs. new buffer. Right: Scatter plot of fragment quantification in peaks (log2) of GM12878 using the new reverse crosslink buffer with cleanup vs. new buffer without any cleanup (Pi-ATAC); **c**, UCSC genome browser tracks of ATAC-seq peaks of fixed GM12878 (green), fixed GM12878 with new reverse crosslink buffer (red) and Pi-ATAC GM12878 peaks (blue); **d**, TSS enrichment of fixed ATAC-seq libraries prepared with old reverse crosslink buffer (green), new reverse crosslink buffer (red) and Pi-ATAC (blue) (all GM12878); **e,** ChromHMM profile of the ATAC-seq peak distribution across the genome. Left two columns show old reverse crosslink buffer, middle the new reverse crosslink buffer, right two columns bulk Pi-ATAC data of GM12878 cells (all replicates).

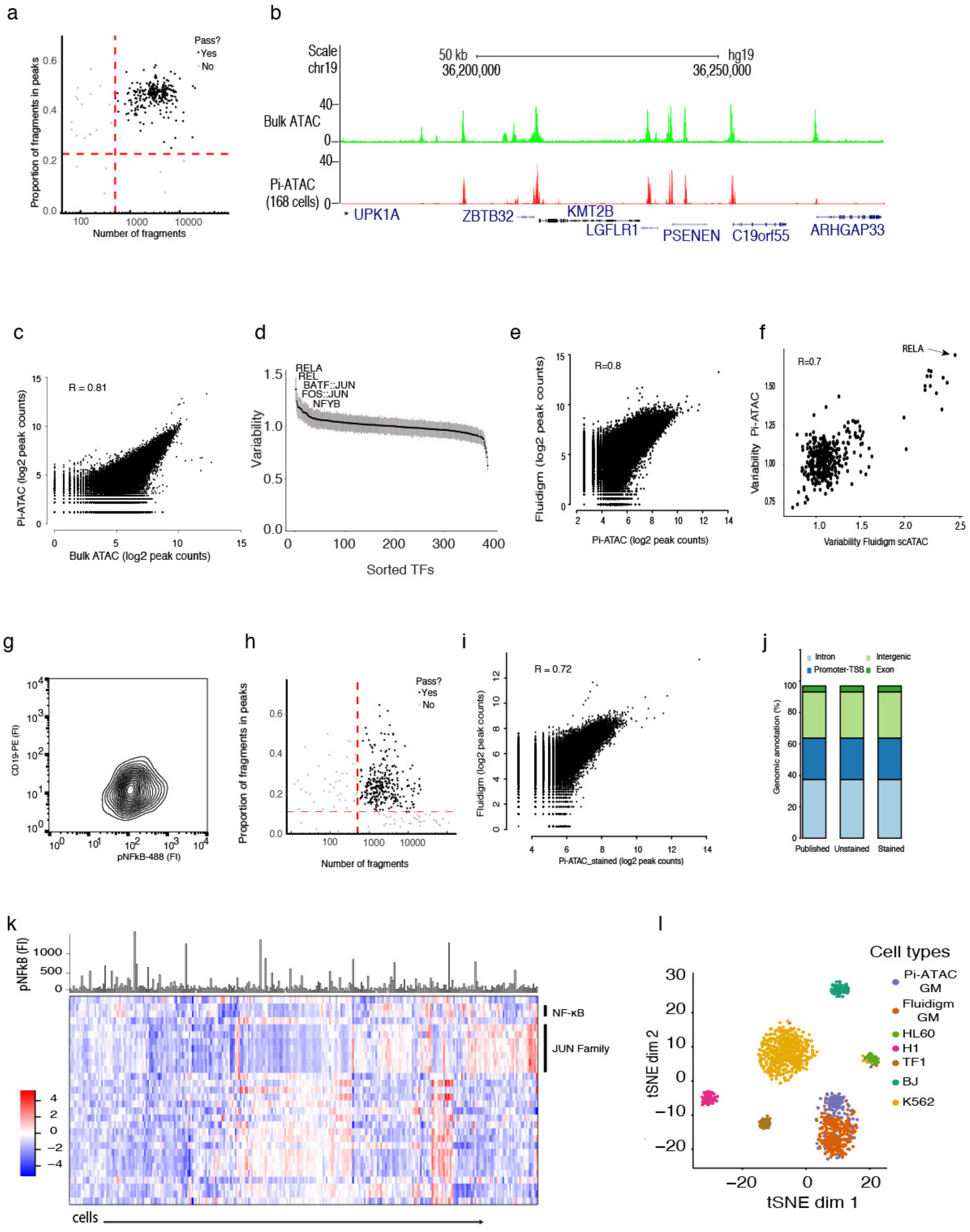

Supplementary Figure 2: Single cell Pi-ATAC of GM12878 cells. **a,** QC of unstained Pi-ATAC GM12878 data: each dot represents a cell, gray cells did not pass the filter of enough fragments and enough proportion of fragments in peaks. 168 of 192 sorted cells (87.5 %) passed the filter and were used for downstream analysis; **b**, UCSC genome browser track comparison of aggregated 168 single cell Pi-ATAC to bulk ATAC-seq (both GM12878). **c**, Scatter plot of fragment quantification in peaks (log2) of published

GM12878 ATAC-seq data to unstained Pi-ATAC GM12878. Correlation was calculated using Pearson correlation coefficient;
d, Ranking of transcription factor (TF) motif variability plot of unstained Pi-ATAC data (GM12878). **e**, Scatter plot of fragment quantification in peaks (log2) of published scATAC-seq GM12878 data to unstained Pi-ATAC GM12878, Pearson correlation coefficient shown;
f, Scatter plot of transcription factor (TF) accessibility variability of published scATAC-seq GM12878 data to motif variability of unstained Pi-ATAC GM12878 (Pearson correlation coefficient); **g,** FACS density plot of GM12878 cells fixed and stained with CD19 and pNFkB; **h**, QC of stained Pi-ATAC GM12878 data: each dot represents a cell, gray cells did not pass the filter of enough fragments and enough proportion of fragments in peaks. 298 of 384 sorted cells (77.6 %) passed the filter and were used for downstream analysis; **i,** Scatter plot of fragment quantification in peaks (log2) of published scATAC-seq GM12878 data compared to stained Pi-ATAC GM12878, Pearson correlation coefficient shown; **j**, Profile of scATAC-seq peak distribution across the genome. Left: published data, Middle: unstained Pi-ATAC data, Right: stained Pi-ATAC data. All libraries were prepared from GM12878 cells; **k**, Unsupervised hierarchical clustering of the transcription factor accessibility deviations (rows) across stained GM12878 cells (columns). Staining intensity of pNF-kB of each cell is shown by the bargraph above the heatmap; **l**, t-SNE projection generated from TF deviation z-scores. Pi-ATAC data of GM12878 cells (violet) were projected together with scATAC-seq of GM12878 cell on Fluidigm platform(orange), and distinct from other cell types[3].

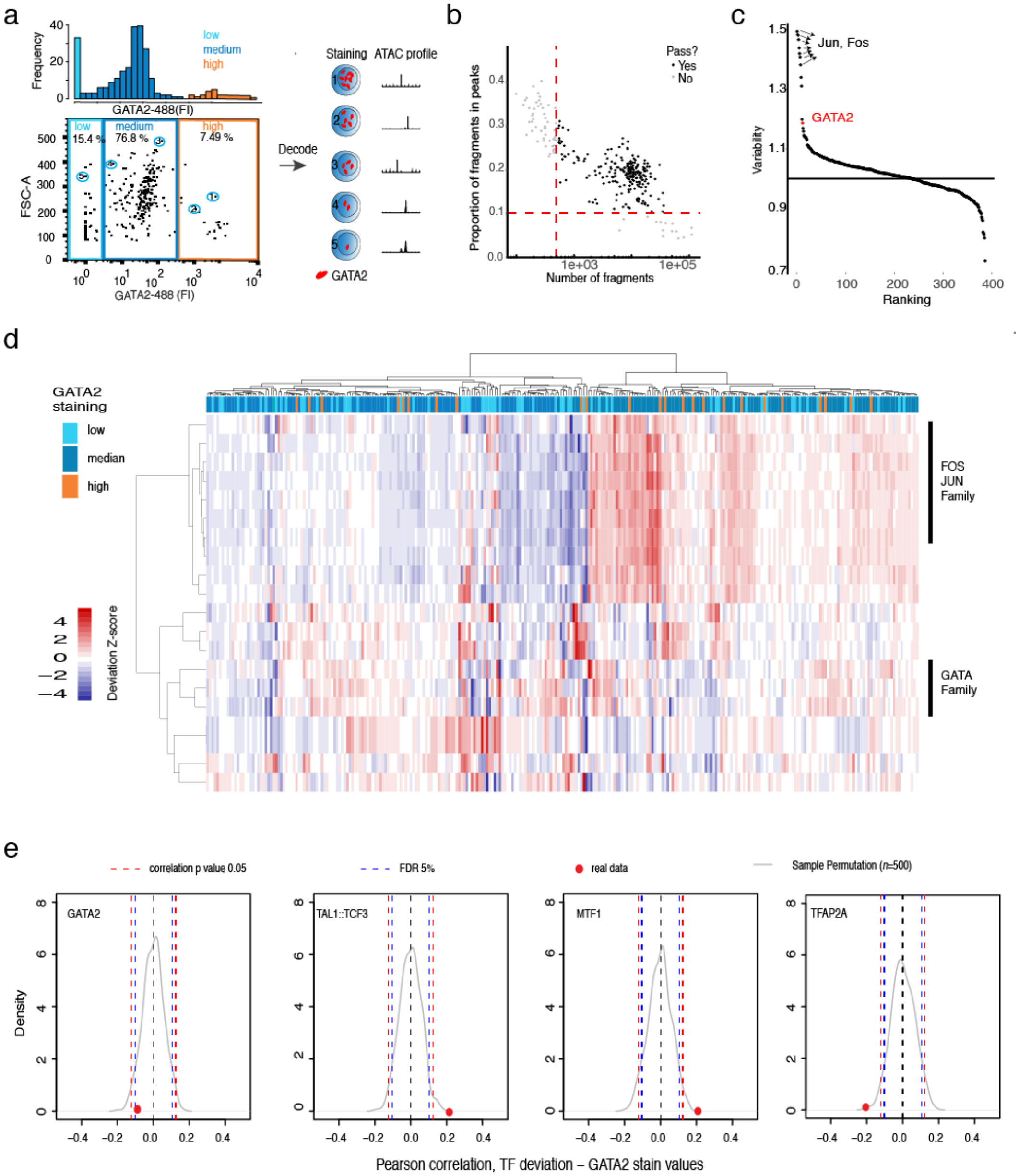

Supplementary Figure 3: Single cell Pi-ATAC of stained K562 nuclei. **a,** Dot plot and histogram of the index sorted 288 K562 nuclei stained for GATA2. Gates indicate low (15.4%), medium (76.8%) and high (7.49%) staining groups; **b**, QC of stained Pi-ATAC K562 nuclei: each dot represents a cell, gray cells did not pass the filter of enough fragments and enough proportion of fragments in peaks. 223 of 288 sorted cells (77.4%) passed the filter and were used for downstream analysis; **c**, Ranking of TF motif variability of K562 Pi-ATAC data; **d**, Unsupervised hierarchical clustering of the transcription factor accessibility deviations (rows) across stained K562 nuclei (columns). Staining intensity of GATA2 of each cell is shown by the color bar above the heat map; **e**, Pearson correlation coefficients of staining intensity with selected transcription factor accessibility deviations (red dots). Left to right: GATA2, TAL::TCF3, MTF1, TFAP2A. The correlation coefficients corresponding to p-value 0.05 are shown by dotted line (red). 500 times of permutation was processed on cell labels with distribution of correlation coefficient shown as density plot (grey curve), and FDR 5% shown by dotted line (blue).

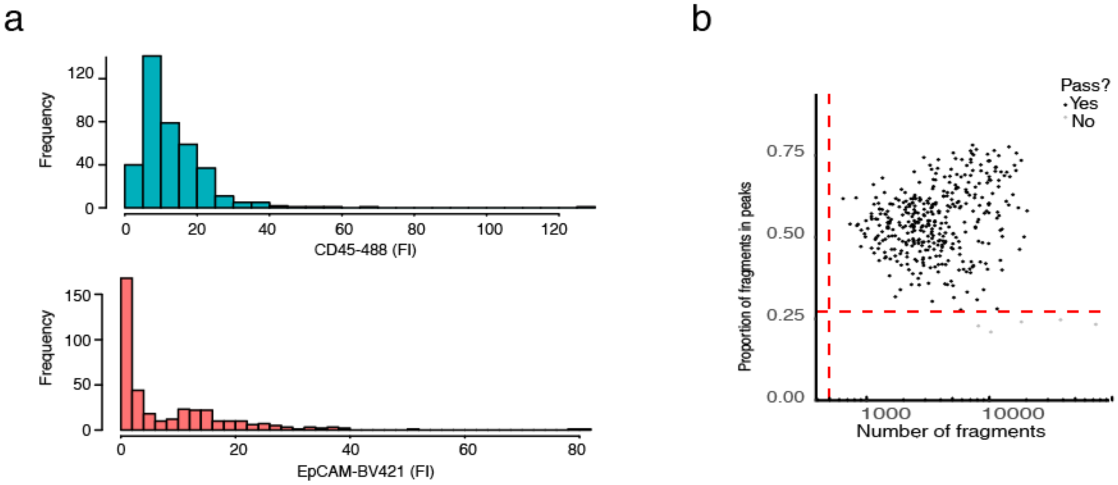

Supplementary Figure 4: Pi-ATAC of a mixture of 4T1 and Splenocytes. **a,** The histogram represents the protein staining of CD45+ cells (upper panel) and EpCAM+ cells (lower panel) from the 4T1-Splenocytes mixture**;** **b**, QC of Pi-ATAC of stained 4T1- Splenocytes mixture. 190 of 192 sorted cells (98.95%) passed the filter and were used for downstream analysis.

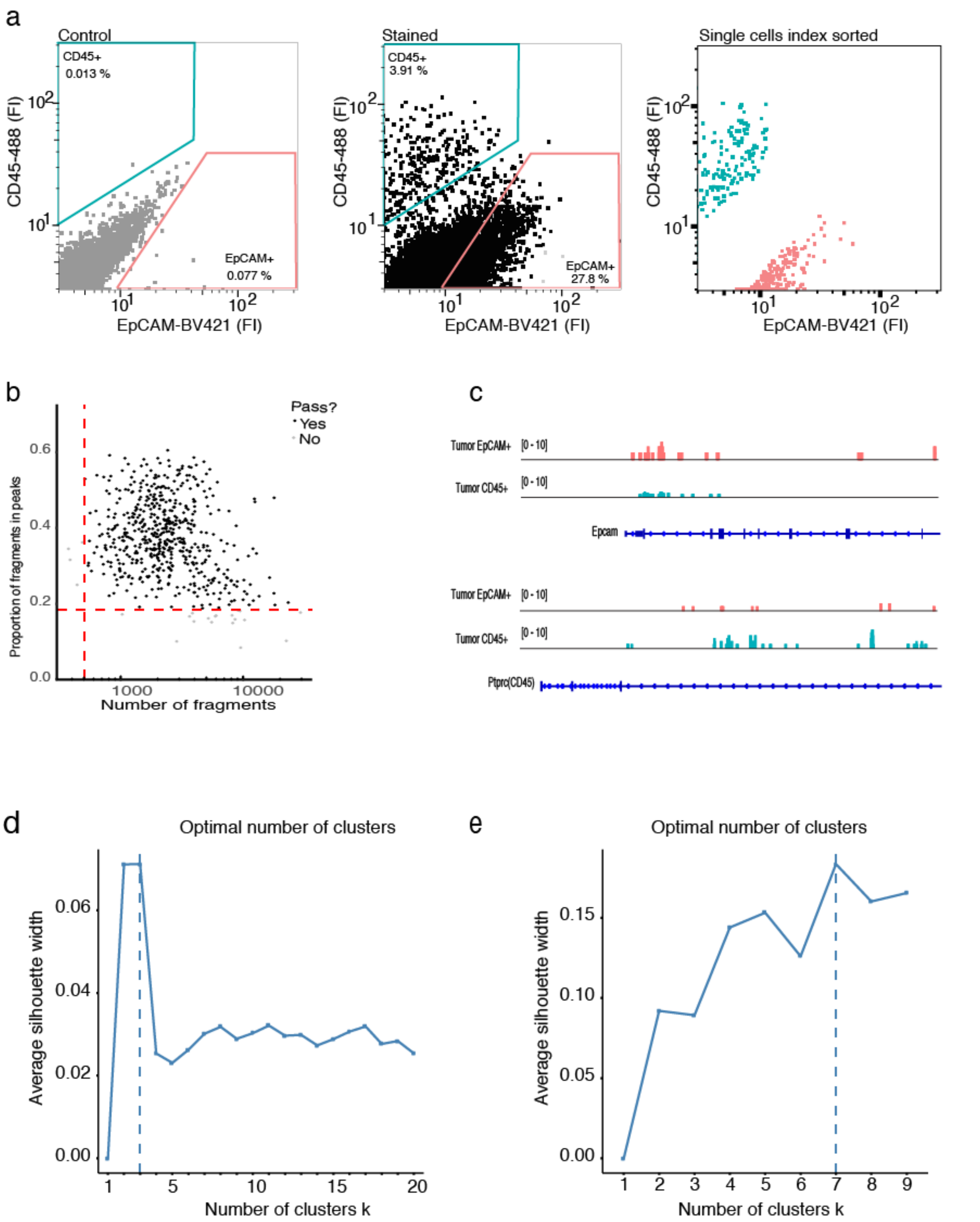

Supplementary Figure 5: Pi-ATAC of tumor and immune cells isolated from a mouse breast tumor. **a,** FACS gating strategy to collect EpCAM+ and CD45+ cells from the same mouse breast tumor (left two plots) and resulting sorted cells (right plot) **b,**QC of Pi-ATAC of EpCAM - and CD45 positive cells isolated from a tumor. 369 of 384 sorted cells (96.09%) passed the filter and were used for downstream analysis; **c,** UCSC genome browser tracks of Pi-ATAC peaks of CD45+ immune cells (green), and EpCAM+ tumor cells (red) at the *Epcam* (top) and *Cd45* locus (bottom). **d-e**, The number of clusters was determined by average silhouette width with R package “factoextra”. The best number of clusters is three for transcription factor motif accessibility (**d**) and seven for cell subgroups (**e**).

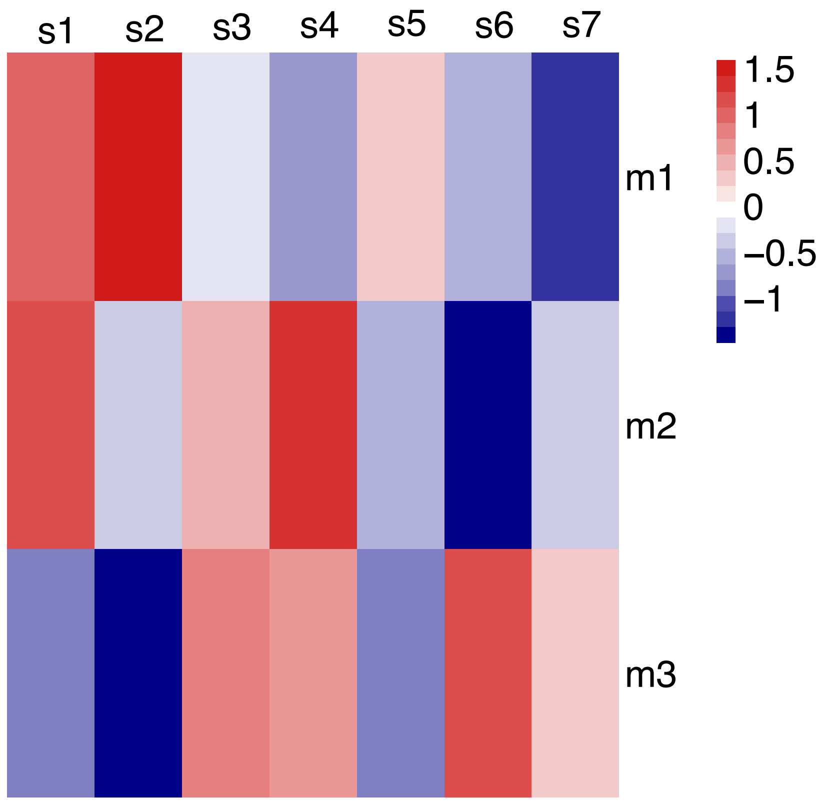

Supplementary Figure 6: Mean of the TF motif deviation within the modules and subcellular clusters indicated (see Figure 3d).

a

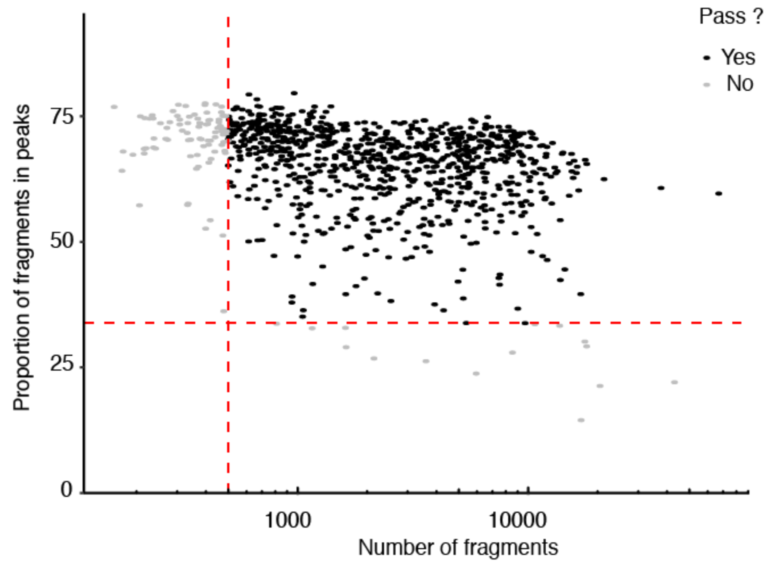

b

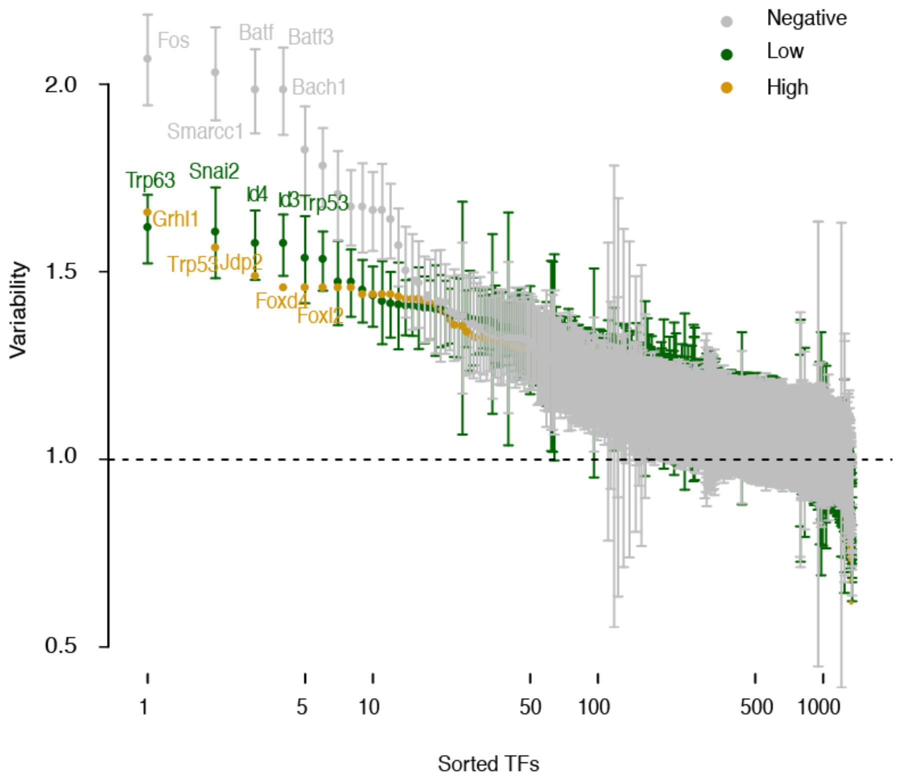

Supplementary Figure 7: Pi-ATAC of EpCAM and HIF1α positive tumor cells isolated from a mouse breast tumor. **a**, QC of Pi-ATAC of EpCAM and HIF1α double positive cells isolated from a tumor. 839 of 956 sorted cells (87.76%) passed the filter and were used for downstream analysis; **b**, transcription factor motif ranking variability plot of Pi-ATAC-seq in different staining groups of HIF1α after downsampling to equal cell numbers (100 times of simulation), mean value and standard deviation shown by error bars.

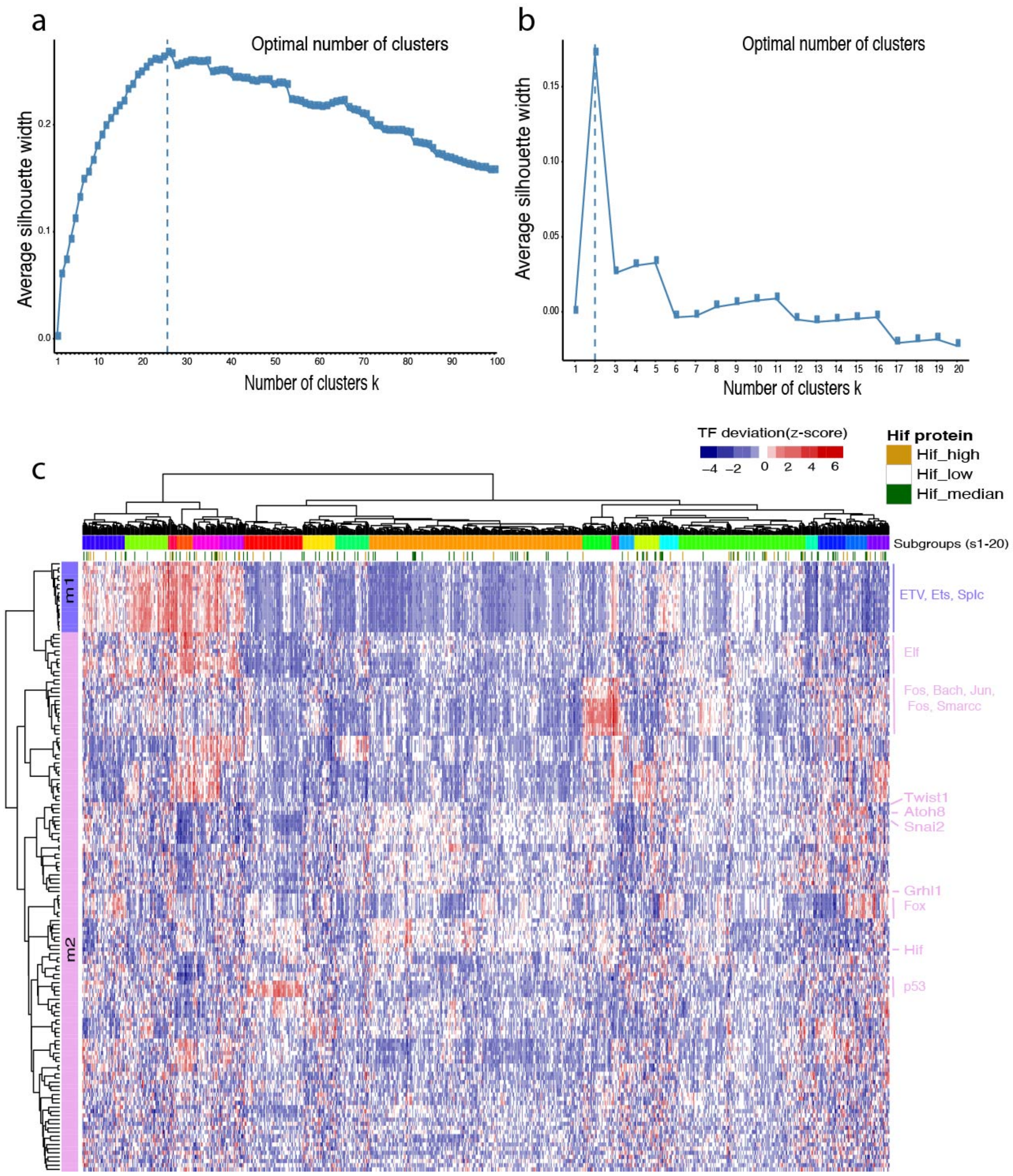

Supplementary Figure 8: Pi-ATAC of EpCAM+ HIF1α+ tumor and immune cells isolated from a mouse breast tumor. **a,b** The number of clusters of the hierarchical cluster was determined by average silhouette width with R package "NbClust". The best number of clusters is two for transcription factor motif accessibility (**a**) and 53 for cell subgroups. Cell subgroups with less than five cells were merged to higher-level nodes, and ended with 20 subgroups (**b**);**c** Unsupervised hierarchical clustering of the TF deviation z-scores of all significant variable TFs ($p < 0.000001$after Benjamini-Hochberg correction on multiple tests) across EpCAM+ HIF1a+ cells from a mouse breast tumor. Each column represents one cell and each row a transcription factor motif. Motif modules (m1-2) and cell subgroups (s1-20) are marked with distinguished colors. In addition, the HIF1α staining information from FACS was assigned to each individual cell (top color bar).

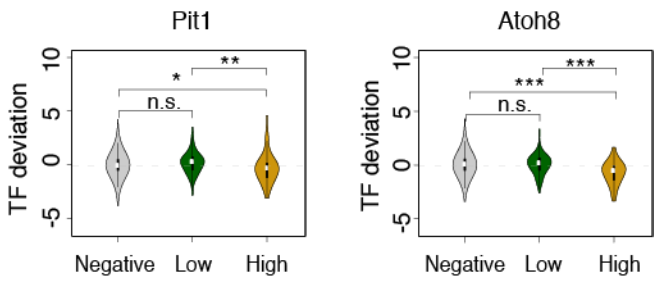

Supplementary Figure 9: Pit1 TF motif deviation across HIF1α staining groups (left) and Atoh8 TF motif deviation across HIF1α staining groups (right). In each comparison, HIF1α negative (*n*=702), HIF1α low (*n*=95), HIF1α high (*n*=42).

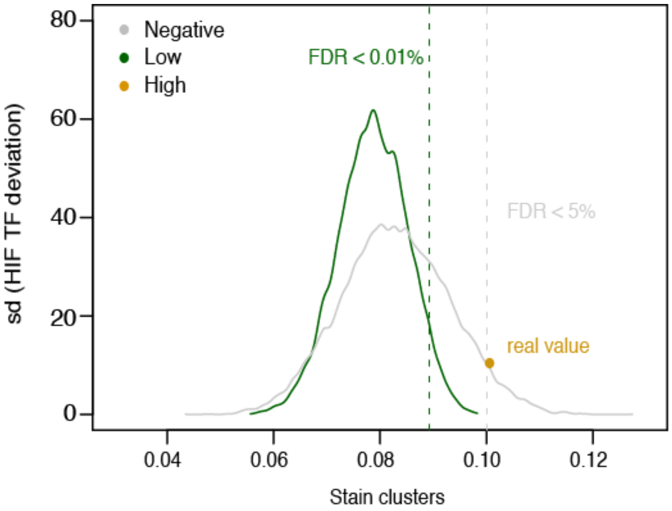

Supplementary Figure 10: Standard deviation of HIF motif accessibility deviation in high HIF1α cluster (yellow dot). 10,000 times of down-sampling simulation in low and median HIF1α clusters to the same number of cells as found in the high HIF1α cluster (*n*=42). Density plot of standard deviation distributions of HIF motif deviation re-calculated in simulation is plotted (green and grey curves) to confirm the slight but significant (FDR < 0.04) higher heterogeneity of HIF motif in high HIF1α cluster compared to median and low HIF1α clusters.

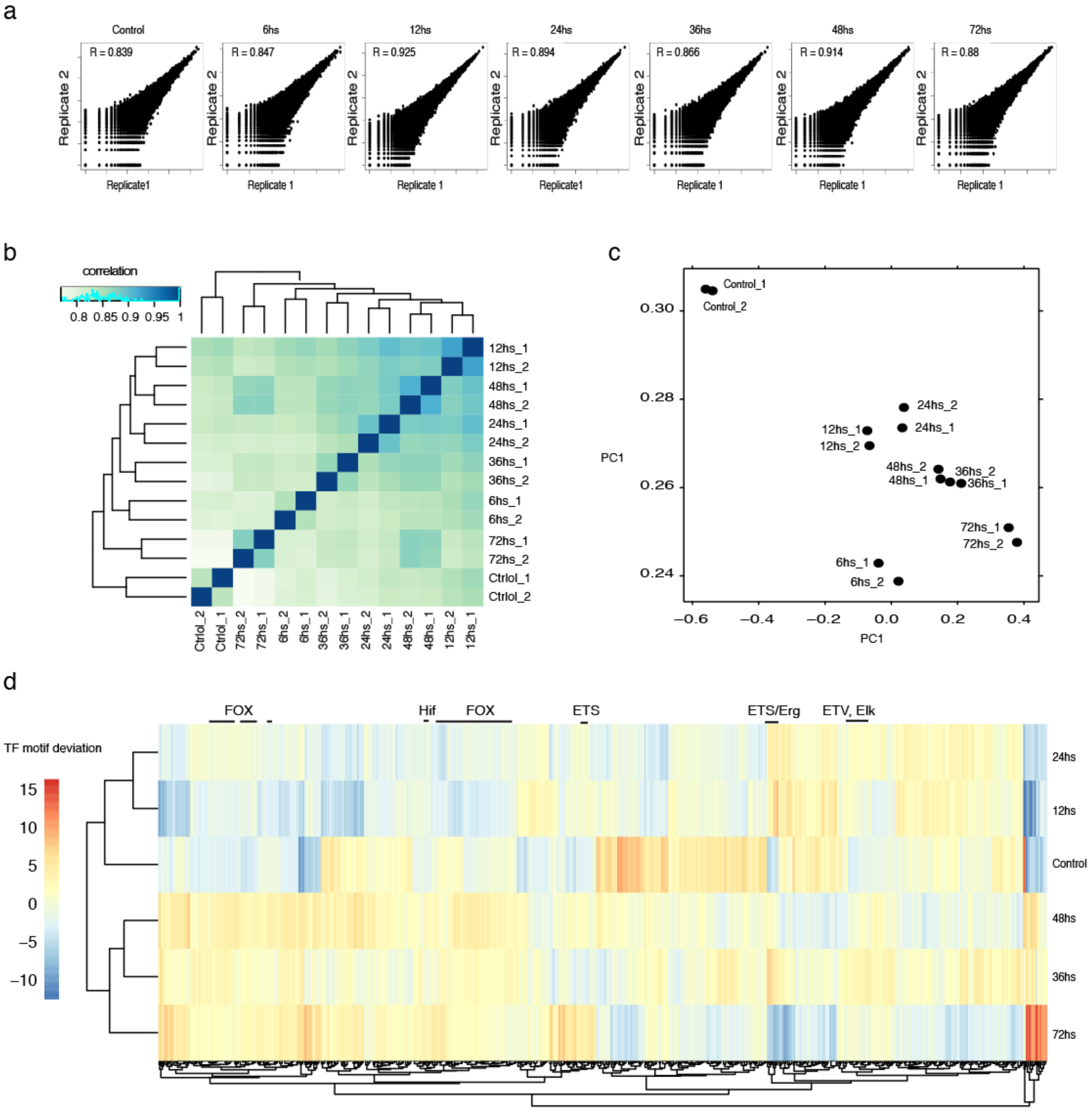

Supplementary Figure 11: The summary of the hypoxia time course ATAC-seq from 4T1. **a**, Reproducibility of ATAC-seq from different time points under hypoxic conditions; **b**, Correlation matrix of all ATAC-seq samples across different time points under hypoxia; **c**, PCA plot shows clustering of chromatin accessibility from different time points under hypoxia condition; **d**, transcription factor motif accessibility across different time points under hypoxic conditions analyzed using chromVAR using the CIS-BP motif database (see **Methods**).

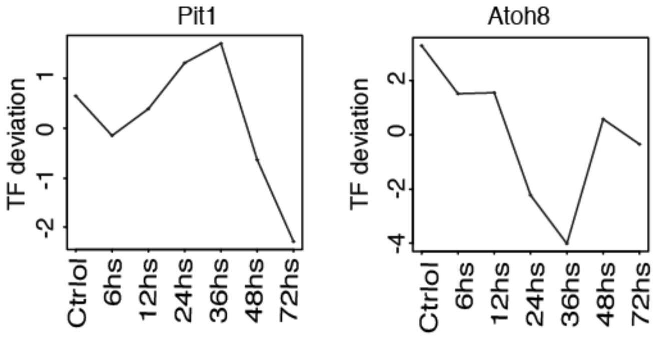

Supplementary Figure 12: Pit1 TF motif accessibility deviation across HIF1α staining groups (left) and Atoh8 TF motif accessibility deviation across HIF1α staining groups (right) in 4T1 cells.

References:

1. Chen, X. et al. ATAC-see reveals the accessible genome by transposase-mediated imaging and sequencing. *Nature methods* **13**, 1013-1020 (2016).
2. Charrad, M., Ghazzali, N., Boiteau, V. & Niknafs, A. Nbclust: An R Package for Determining the Relevant Number of Clusters in a Data Set. *J Stat Softw* **61**, 1-36 (2014).
