# Supplementary material for: Joint single-cell DNA accessibility and protein epitope profiling reveals environmental regulation of epigenomic heterogeneity
Source: Nat Commun. 2018 Nov 2;9:4590. doi: 10.1038/s41467-018-07115-y (PMC6214962; doi:10.1038/s41467-018-07115-y)
Supplement: Supplementary file 3 — Description of Additional Supplementary Files [file 41467_2018_7115_MOESM3_ESM.pdf]

Description of Additional Supplementary Files

File Name: Supplementary Data 1
Description: List of the TF motif variability of the EpCAM+ and CD45+ cells isolated from a mouse tumor calculated by chromVAR.

File Name: Supplementary Data 2
Description: List of the significant variable motifs and their module assignment from the EpCAM+ and CD45+ cells isolated from a mouse tumor.

File Name: Supplementary Data 3
Description: List of the significant 6-mers from the EpCAM+ and CD45+ cells isolated from a mouse tumor.

File Name: Supplementary Data 4
Description: List of the significant 7-mers from the EpCAM+ and CD45+ cells isolated from a mouse tumor.

File Name: Supplementary Data 5
Description: List of the significant 8-mers from the EpCAM+ and CD45+ cells isolated from a mouse tumor.

File Name: Supplementary Data 6
Description: List of the 755 significant (p-value < 0.05, B.H.) TF motif variability of the 4T1 cells across a hypoxia time course (calculated by chromVAR).

File Name: Supplementary Data 7
Description: List of the differential TF motif deviations of EpCAM+ and HIF1A+ cells, including statistical tests for significance across the 3 staining groups.

File Name: Supplementary Data 8
Description: List of the TF motif variability of EpCAM+ and HIF1+cells isolated from a mouse tumor, calculated by chromVAR.
